# Congenital duodenal obstruction repair with and without transanastomotic tube feeding: a systematic review and meta-analysis

**DOI:** 10.1136/archdischild-2023-325988

**Published:** 2023-11-03

**Authors:** George Stephen Bethell, Jonathan J Neville, Mark John Johnson, Joanne Turnbull, Nigel J Hall

**Affiliations:** 1 University Surgery Unit, Faculty of Medicine, University of Southampton, Southampton, UK; 2 NIHR Southampton Biomedical Research Centre, University of Southampton and University Hospital Southampton NHS Foundation Trust, Southampton, UK; 3 Department of Neonatal Medicine, University Hospital Southampton NHS Foundation Trust, Southampton, UK; 4 School of Health Sciences, Faculty of Environmental and Life Sciences, University of Southampton, Southampton, UK

**Keywords:** Neonatology, Gastroenterology

## Abstract

**Objective:**

To determine the impact of transanastomotic tube (TAT) feeding in congenital duodenal obstruction (CDO).

**Design:**

Systematic review with meta-analysis.

**Patients:**

Infants with CDO requiring surgical repair.

**Interventions:**

TAT feeding following CDO repair versus no TAT feeding.

**Main outcome measures:**

The main outcome was time to full enteral feeds. Additional outcomes included use of parenteral nutrition (PN), cost and complications from either TAT or central venous catheter. Meta-analyses were undertaken using random-effects models (mean difference (MD) and risk difference (RD)), and risk of bias was assessed using the Risk Of Bias In Non-randomised Studies - of Interventions (ROBINS-I) tool.

**Results:**

Twelve out of 373 articles screened met the inclusion criteria. All studies were observational and two were prospective. Nine studies, containing 469 infants, were available for meta-analysis; however, four were excluded due to serious or critical risk of bias. TAT feeding was associated with reduced time to full enteral feeds (−3.34; 95% CI −4.48 to −2.20 days), reduced duration of PN (−6.32; 95% CI −7.93 to −4.71 days) and reduction in nutrition cost of £867.36 (95% CI £304.72 to £1430.00). Other outcomes were similar between those with and without a TAT including inpatient length of stay (MD −0.97 (−5.03 to 3.09) days), mortality (RD −0.01 (−0.04 to 0.01)) and requirement for repeat surgery (RD 0.01 (−0.03 to 0.05)).

**Conclusion:**

TAT feeding following CDO repair appears beneficial, without increased risk of adverse events; however, certainty of available evidence is low. Earlier enteral feeding and reduced PN use are known to decrease central venous catheter-associated risks while significantly reducing cost of care.

**PROSPERO registration number:**

CRD42022328381.

**WHAT IS ALREADY KNOWN ON THIS TOPIC**

- Transanastomotic tube feeding following congenital duodenal obstruction repair has been shown to be beneficial in individual studies.

**WHAT THIS STUDY ADDS**

- Pooled data show that transanastomotic tube insertion allows earlier full enteral feeds and reduced parenteral nutrition use.

**HOW THIS STUDY MIGHT AFFECT RESEARCH, PRACTICE OR POLICY**

- Further understanding of barriers to use of transanastomotic tubes is required in order to promote use and improve outcomes in infants with congenital duodenal obstruction.

## Introduction

Congenital duodenal obstruction (CDO), comprising the conditions duodenal atresia and duodenal web, with or without an annular pancreas, has a reported incidence of 1 in 8200 live births and is a condition for which surgical repair is usually undertaken within the first few days of life.^1^ Following repair, there is variation in the nutritional management of these infants.^2^ Nutritional support is required since although continuity of the gastrointestinal tract has been surgically restored, there remains a functional obstruction due to many months of in-utero obstruction resulting in dilatation of the stomach and proximal duodenum, with gastroparesis. Existing data demonstrate that current nutritional management usually comprises provision of parenteral nutrition (PN), provision of enteral nutrition via a transanastomotic tube (TAT) or a combination of both.^2^ TAT insertion at time of CDO repair is an effective method of allowing provision of early enteral nutrition and carries several purported advantages that have prompted its uptake by some clinicians. These include reduced use of PN, associated reduced requirement for central venous access, earlier achievement of full enteral feeds and reduced cost.^3–5^ However, others prefer not to use a TAT for feeding, citing concerns about tube-related small bowel perforation^4^ and pointing out that despite earlier achievement of full enteral feeds, TAT feeding does not reduce length of stay since this is dependent on recovery of gastric function and TAT feeding does not impact this.^2 6^

Understanding the optimal nutritional management of these infants may result in improved patient outcomes, improved clinician and carer satisfaction and may reduce cost. As such, there is ongoing interest in feeding modalities after CDO repair. A systematic review and meta-analysis published in 2021 including six retrospective studies found that TAT feeding was associated with a reduction of 6.63 days to full enteral feeds, while PN use was reduced by 5.38 days compared with those where TAT feeding was not used.^7^ The authors reported that this study was limited by its retrospective nature and 50% of studies had serious or critical concerns of bias due to confounding.

Recently, there have been further publications in this field including the first multicentre prospective study comparing outcomes of TAT versus no TAT placement in CDO and another large retrospective study addressing this research question.^2 5^ Given this vast increase in cases reported over the previous 3 years, we aimed to provide an up-to-date comparison of outcomes of CDO repair with and without TAT feeding.

## Methods

This study was designed and carried out as recommended by the Preferred Reporting Items for Systematic Reviews and Meta-Analyses guidance.^8^ The protocol was registered on PROSPERO (CRD42022328381) on 9 May 2022. For further details on methods, see online supplemental materials.

10.1136/archdischild-2023-325988.supp1 Supplementary data

### Literature search and study selection

Medline, CINAHL, Embase and the Cochrane Library were searched on 6 March 2023. Case reports of complications related to TAT were also sought. Studies were included if they compared use of TAT feeding in CDO versus no TAT.

### Data extraction and bias assessment

Studies with duplicate participants and those containing no comparative outcomes were excluded from quantitative synthesis. Bias was assessed using the Risk Of Bias In Non-randomised Studies - of Interventions (ROBINS-I) tool and studies with serious or critical risk of bias were excluded at the quantitative analysis stage. Risk of bias plots were created using the open source robvis tool.^9^

### Outcomes

The main outcome was time to full enteral feeds. Additional outcomes were time to full pre-anastomotic feeds, time to full oral feeds, duration of PN, length of inpatient stay, cost of nutrition, mortality, sepsis, reoperation and complications of TAT or central venous catheter (CVC)/PN. CVC-related sepsis was included as a CVC/PN complication and sepsis.

## Results

### Qualitative analysis

**Figure 1:**
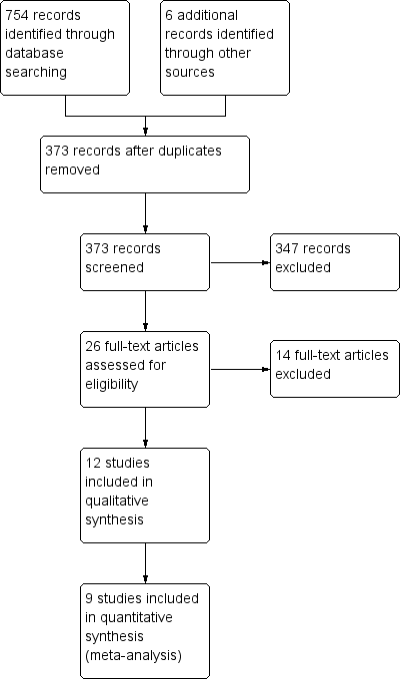
Preferred Reporting Items for Systematic Reviews and Meta-Analyses flow diagram.

There were 12 studies^2–6 10–16^ that met the inclusion criteria (figure 1), all of which were observational. Of these, two were prospective^2 11^ while the remainder were retrospective (table 1). An overview of outcomes provided in included studies, where a statistical comparison between TAT and no TAT use has been made, is shown in table 2. In summary, most studies (seven of eight) that reported time to starting enteral feeds found that this was shorter with TAT use. TAT use was also found to be beneficial in five of eight studies that reported PN duration and five or six studies that reported CVC use. One study reported that TAT use allows earlier commencement of pre-anastomotic feeds and another found that there is lower nutrition-related cost associated with TAT use.

**Table 1:** Summary of the 12 included studies

Study	Study design	Infants (n)		Birth weight (kg)		Outcomes reported	Excluded from quantitative synthesis
		TAT	No TAT	TAT	No TAT		
Arnbjörnsson *et al* 10	Retrospective	16	9	2.8^$^	2.17^$^	Time to starting enteral feeds, time to starting pre-anastomotic feeds, time to full pre-anastomotic feeds, CVC requirement, CVC duration, PN requirement, PN duration, length of stay, sepsis	
Aroonsaeng *et al* 11	Prospective	7	6	1.95	2.22	Time to starting enteral feeds, time to full enteral feeds, time to full pre-anastomotic feeds, CVC requirement, CVC duration, PN duration, length of stay, complications including TAT or CVC associated	
Bairdain *et al* 12	Retrospective	13	74	–	–	Duration of PN, time to full enteral feeds (both without data for no TAT group) and anastomotic leak rate	Yes
Bethell *et al* 2	Prospective	43	59	2.35	2.5	Time to starting enteral feeds, time to full enteral feeds, CVC requirement, PN requirement, PN duration, length of stay, complications included TAT or CVC associated, mortality, standardised weight change from birth to 28 days, standardised weight at 1 year	
Cresner *et al* 6	Retrospective	54	42	2.71	2.57	Time to starting enteral feeds, time to full enteral feeds, time to full pre-anastomotic feeds, CVC requirement, PN requirement, PN duration, length of stay, complications included TAT or CVC associated, mortality	
Hall *et al* 3	Retrospective	17	38	2.8	2.6	Time to starting enteral feeds, time to full enteral feeds, time from starting to full enteral feeds, CVC requirement, PN requirement, length of stay, complications included TAT or CVC associated, mortality	Yes
Harwood *et al* 4	Retrospective	37	22	2.85	2.64	Time to starting enteral feeds, time to full pre-anastomotic feeds, CVC requirement, PN requirement, PN duration, cost of PN, cost of nutrition (enteral feeds and PN), TAT complications	
Mooney *et al* 13	Retrospective	10	9	–	–	Time to starting TAT feeds, time to starting oral feeds, time to discontinuation of IV fluids, length of stay	
Ruangtrakool *et al* 14	Retrospective	4	30	2.19^$^	2.2^$^	Time to starting enteral feeds, time to full enteral feeds, PN requirement, PN duration, length of stay, complications, sepsis, mortality	
Treider *et al* 5	Retrospective	37	63	2.84^$^	2.50^$^	Time to starting enteral feeds, time to breast feeding, breast feeding started before discharge, time to full pre-anastomotic feeds, CVC requirement, PN duration (at authors’ institution), PN requirement at discharge, length of stay (at authors’ institution), complications included TAT or CVC associated, growth (g/day), mortality	
Treider *et al* 15	Retrospective	88	98	–	–	PN duration, length of stay, complications included TAT or CVC associated, mortality	Yes
Upadhyay *et al* 16	Retrospective	12	9	2.6^$^	2.6^$^	Time to full oral feeds, PN requirement, PN duration, length of stay, mortality	

Birth weight is median unless identified by $, in which case, it is mean.

CVC, central venous catheter; IV, intravenous; PN, parenteral nutrition; TAT, transanastomotic tube.

**Table 2:** Summary of outcomes reported from qualitative synthesis

	Better with TAT	No difference	Worse with TAT
Starting oral feeds			1 study^16^
Full oral feeds		1 study^13^	
Starting pre-anastomotic feeds	1 study^10^		
Full pre-anastomotic feeds	1 study^10^	4 studies^4–6 11^	
Starting enteral feeds	7 studies^2–6 10 11^	1 study^14^	
Full enteral feeds	2 studies^3 6^	3 studies^2 12 14^	1 study^11^
PN duration	5 studies^2 4–6 15^	3 studies^11 12 14^	
CVC use	5 studies^2 3 5 6 10^	1 study^4 11^	
Nutrition cost	1 study^4^		
Length of stay		6 studies^2 3 5 6 11 14^	3 studies^13 15 16^
Standardised weight change		2 studies^2 5^	
Complication rate		4 studies^2 11 12 15^	1 study^5^

All outcomes are considered better if time is shorter or use is less except from standardised weight change.

Outcome only included in this table if statistical comparison between TAT and no TAT group has occurred.

CVC, central venous catheter; PN, parenteral nutrition; TAT, transanastomotic tube.

The majority of studies (four of five) found similar times to full pre-anastomotic feeds in those with and without a TAT and similar length of stay was reported in six of nine studies. Four out of five studies that reported a comparison of complication rates found these were also similar between TAT and no TAT use. The only two studies that reported standardised weight change and the only study that reported time to full oral feeds also found similar outcomes with and without TAT use.

One study reported that time to starting oral feeds was longer with a TAT, while one of six reported longer duration to full enteral feeds. Length of stay was found to be longer in three of nine studies with a TAT, and one of five studies found a higher complication rate with TAT versus without.

### Risk of bias

**Figure 2:**
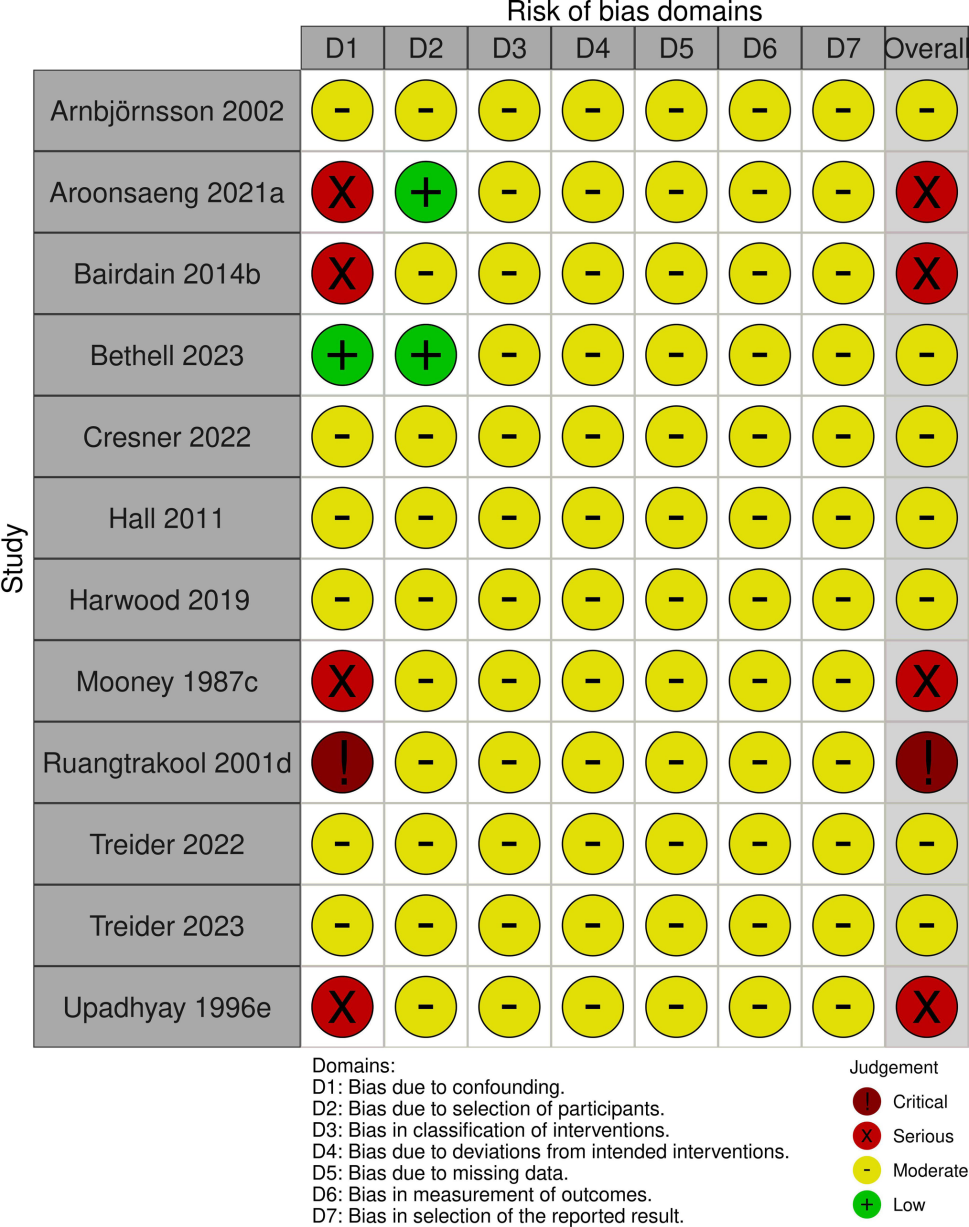
Risk of bias assessment using Risk Of Bias In Non-randomised Studies - of Interventions (ROBINS-I) tool. (a) Serious risk of confounding due to small group size and significant difference in gestational age and weight between groups. (b) Serious risk of confounding due to large difference between group size and no information regarding demographics of each group as not primary aim of study. (c) Serious risk of confounding as six infants received a gastrostomy and it is unclear which group they belong to. (d) Critical risk of confounding as transanastomotic tube group only contains four infants and two of these received a gastrojejunostomy rather than nasojejunostomy. (e) Serious risk of confounding due to small group size and transanastomotic tube passed via gastrostomy rather than nasojejunostomy.

Given the retrospective nature of most studies, risk of bias was assessed predominantly as moderate (figure 2). Five studies had serious or critical risk of bias due to confounding which was reflected in the overall bias assessment (figure 2).

### Study selection for quantitative synthesis

Of the 12 included studies, 2 contained a duplicate population of patients. All of the infants included in Hall *et al* 3 were also included in Cresner *et al,* 6 so data from the most recent study were used for quantitative synthesis. Another study, Treider *et al*,^15^ contained a population of infants that overlapped with Treider *et al*.^5^ Given the primary aim of the study from 2022 was to compare TAT use with no TAT use and the most recent study^15^ contained few data on this outcome, the study from 2022^5^ was used for quantitative synthesis. A further study by Bairdain *et al* 12 contained no comparative data on prespecified outcomes so was also not included in quantitative analysis. This left nine studies reporting in total 469 infants available for quantitative synthesis (table 1), all of which were included.

### Quantitative analysis

In the nine studies, 220 (46.9%) infants had a TAT and 249 (53.1%) did not have a TAT inserted at CDO repair. Studies found to have serious or critical risk of bias (figure 2) were excluded from quantitative synthesis.

#### Continuous variables

**Figure 3:**
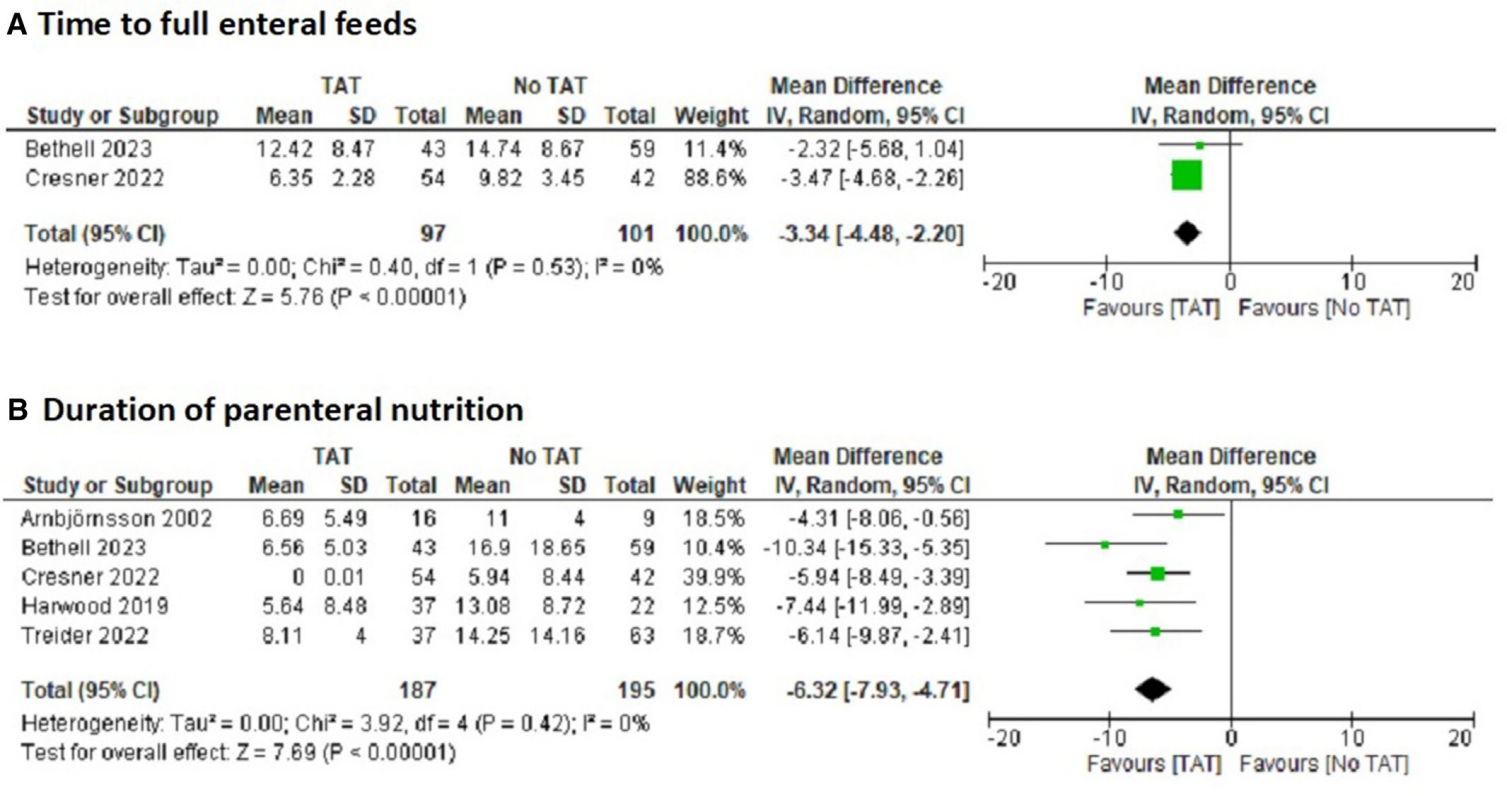
Forest plots showing meta-analyses: (A) time to full enteral feeds, (B) duration of parenteral nutrition. TAT, transanastomotic tube.

TAT use was associated with 3.34 (95% CI 2.2 to 4.48) fewer days to full enteral feeds (figure 3A), 6.32 (95% CI 4.71 to 7.93) fewer days of PN (figure 3B) and a reduction in nutrition cost of £867.36 (95% CI £304.72 to £1430.00). All other continuous variable outcomes were found to be similar between TAT and no-TAT groups on pooled analysis (table 3).

**Table 3:** Outcomes of transanastomotic tube (TAT) use versus no TAT excluding studies with serious or critical risk of bias

Outcome	Studies	Infants	Mean difference or risk difference (95% CI)
Time to full enteral feeds	2	198	**−3.34 (–4.48 to –2.20) days**
Time to full pre-anastomotic feeds	4	280	−3.02 (–6.35 to 0.31) days
Time to full oral feeds	0	0	Not estimable
Duration of parenteral nutrition	5	382	**−6.32 (–7.93 to –4.71) days**
Inpatient length of stay	4	323	−0.97 (–5.03 to 3.09) days
Cost of nutrition	1	59	**−£867.36 (–£1430.00 to **–**£304.72**)
Mortality	5	382	−0.01 (–0.04 to 0.01)
Sepsis	4	280	−0.04 (–0.13 to 0.05)
Repeat surgery required	3	298	0.01 (–0.03 to 0.05)
Central venous catheter/parenteral nutrition complication	4	357	−0.09 (–0.22 to 0.04)

Statistically significant findings are in bold font.

#### Complications

Six studies^2–6 11^ reported complications related to CVC and PN. When reported, these complications consisted of CVC-associated sepsis,^3–6^ displacement,^6^ extravasation^3 6^ and localised site swelling.^6^ In meta-analysis, rates of CVC/PN complications were similar between those with and without a TAT (table 3). Similarly, rate of mortality and need for further surgery were similar between groups.

Five studies^3–6 11^ reported TAT-related complications, the most clinically important of which is anastomotic leak or small bowel perforation which was reported in two studies. Cresner *et al* 6 reported anastomotic leak in a single infant following CDO repair with TAT placement who subsequently was found to have Hirschsprung’s disease, and Treider *et al* 5 5 observed an anastomotic leak in an infant where a TAT was used. No study reported small bowel perforation secondary to a TAT. No case reports were identified which reported TAT-related anastomotic leak or perforation in CDO. The overall rate of anastomotic leak associated with TAT, including all those with a TAT in the reported literature not duplicated, was 0.86% (2 of 233; 95% CI 0.00 to 2.04%). Other TAT-related complications were tube displacement or migration,^3–5 11^ tube blockage^5^ and nasal pressure damage from TAT bridle.^6^ On one-sided meta-analysis, the incidence of TAT-related complications was 20% (95% CI 6.1% to 34.0%); however, the majority of these (21 of 24) were tube blockage, dislodgement or migration.

### Certainty of evidence

Grades of Recommendation, Assessment, Development and Evaluation guidelines^17^ determined the certainty of evidence where meta-analysis found a statistically significant difference in outcome with use of TAT versus no TAT (table 4). The certainty of evidence was low due to study design and risk of bias. No evidence included was downgraded.

**Table 4:** Summary of findings with certainty of evidence

Outcome	Number of infants	Anticipated absolute effects (95% CI)	Certainty of evidence
Duration of parenteral nutrition	382	6.32 (4.71 to 7.93) fewer days with TAT	Low*
Time to full enteral feeds	198	3.34 (2.2 to 4.48) fewer days with TAT	Low*
Cost of nutrition	59	£867.36 (£304.72 to £1430.00) less with TAT	Low*

*Evidence was deemed low due to study design and risk of bias.

TAT, transanastomotic tube.

## Discussion

In this systematic review of TAT use in CDO that contains data on over 700 infants, we identified 12 studies, the majority of which concluded that there was a benefit to TAT feeding. When meta-analysis was feasible (up to 382 infants), we found benefit in reduced time to achieve full enteral feed, reduced duration of PN and reduced cost when a TAT is used. There was neither evidence of benefit nor harm in other outcomes compared. Reassuringly, rate of reoperation in both groups was similar which includes potentially significant complications that may be associated with TAT use.

The rationale for TAT use in CDO is to allow enteral feeding beyond the part of the intestine which has been chronically obstructed since early fetal life and beyond the site of surgical repair. Although the mechanical obstruction is relieved by surgical repair, a physiological obstruction remains due to gastroparesis which takes a number of days to resolve. This likely explains why TAT use has not been found consistently to reduce time to full pre-anastomotic or oral feeds and has no impact on duration of hospital stay.^2 6 14^ Length of stay is commonly regarded as an important outcome for surgeons across a range of surgical conditions which could explain why TATs, despite other reported benefits, have not been universally adopted (used in 42–47% of CDO repairs in population-level studies).^2 15^ Yet, our meta-analysis demonstrates there is benefit for the infant and their families for other outcomes including reduced PN requirement and reduced time to full enteral feeds. Our data suggest there may be a very small ‘cost’ to realising these benefits in terms of a very small risk of clinically important TAT-related complications.

Our findings are similar to those reported by a previous systematic review which included fewer studies and participants.^7^ Furthermore, this systematic review is the first to include prospectively collected data. Consequently, these data provide a higher degree of certainty of findings compared with previous work, yet we recognise that due to the design of the included studies and inherent risk of bias, the overall certainty of our findings remains low. This has prompted calls by some to consider the need for well-designed randomised controlled studies to answer this question.^4 7^

Interestingly, both our meta-analysis and the previous smaller one identified fewer benefits to use of a TAT than has been reported by some single-centre studies. We also note that some studies included in this systematic review reported better outcomes than others and there was heterogeneity between studies in effect sizes seen. Overall, single-centre studies^3–6^ tended to show greater benefit to TAT use than multicentre studies^2 15^ and also found benefit across a wider range of outcomes. For instance, some single-centre studies found lower use of CVC and PN in addition to the reduced time to full feeds and time on PN. Cresner *et al* 6 reported a requirement for a CVC in 15% of infants treated with a TAT, whereas in a multicentre population-based study^2^ from the same country, 80% of infants who received a TAT also had a CVC. Our findings suggest that there are differences in practice between centres that likely confound the overall results of multicentre analyses and any meta-analysis; the interventions are likely being delivered in a different way across centres and studies. We suspect that single centres are more likely to have established feeding protocols that guide the use of a TAT, CVCs and PN, whereas multicentre reports encompass a range of different practices for each of these components. This also means that some of the potential benefits of TAT feeding may not be being realised. For instance, it is likely that many infants who have a TAT placed are being unnecessarily exposed to the risks associated with a CVC and PN and not realising the maximum benefit of the TAT.^2^ Established protocols at centres using TATs in CDO, agreed by surgeons, neonatologists and dietitians, might improve this situation.

While some clinicians may not regard the need for a CVC or PN as adequately important to change practice, harms related to treatments, including use of CVCs, are a particular concern to parents of infants.^18^ It has been shown that CVC-related complications occurred in one of four infants in this patient population.^2^ Without the use of a CVC, it is possible to avoid this risk altogether in CDO. It is clear that TAT use can facilitate avoidance of CVC insertion in most of these infants.^4 6^ A further outcome of importance to healthcare providers is the cost of treatment. Only one study reported this; Harwood *et al* found that TAT use resulted in significantly lower treatment costs due to reduced use of PN.^4^

Many of those that do not advocate TAT use in CDO are concerned about tube-related perforation or anastomotic leak. Anastomotic leak was only seen in two infants of which one had an undiagnosed distal bowel obstruction and small bowel perforation was not reported in any included study. Other less severe TAT-related complications including tube dislodgement, blockage and migration were found to have been reported in papers included in this systematic review and overall were seen in 20% of infants with a TAT. These may be avoidable with better securement of the tube externally but even if they do occur are not associated with worse outcomes than not using a TAT initially and usually have negligible impact on the infant. Reporting of timing of these complications was inconsistent; however, one study^5^ reported that unplanned TAT removal, secondary to dislodgement or blockage, occurred 6 days (median) after CDO repair. Even if early TAT removal occurred, these infants will still have been placed in the TAT group in studies, meaning that the benefits of TAT may be under-reported. Future studies should undertake subgroup analysis excluding those where early TAT removal occurs.

In conclusion, TAT use in CDO is associated with earlier full enteral feeds, reduced use of PN and less cost with rarely reported significant complications with the caveat that certainty of evidence was assessed as low given the quality of available studies. Nevertheless, in our opinion, these represent benefits that are likely to justify the use of a TAT. Given the lack of widespread uptake of TATs in this population,^1^ we believe there is scope for further research to understand barriers to TAT implementation. While randomised controlled trials are considered the gold-standard means of testing a clinical intervention, this may not necessarily be required to study this research question. Observational data including this meta-analysis have shown benefit to TAT use, and it is a cheap intervention with rarely reported risk. Additionally, clinicians who routinely use TATs may be unwilling to consider randomisation of their patient to not receive a TAT and hence require a CVC and PN. Heterogeneity of outcomes between studies suggests there is likely patient benefit to be gained from standardisation of treatment pathways for infants with CDO, to include avoidance of CVC and PN when using TAT feeding unless special circumstances (eg, prematurity) exist.

## Data Availability

Data are available upon reasonable request. Database extracted from published articles available by request.
