# Supplementary material for: Congenital duodenal obstruction repair with and without transanastomotic tube feeding: a systematic review and meta-analysis
Source: Arch Dis Child Fetal Neonatal Ed. 2023 Nov 3;109(2):182–8. doi: 10.1136/archdischild-2023-325988 (PMC10894825; doi:10.1136/archdischild-2023-325988)
Supplement: Supplementary data [file fetalneonatal-2023-325988supp001.pdf]

Congenital Duodenal Obstruction Repair with and without Trans-Anastomotic Tube Feeding – A Systematic Review and Meta-Analysis

George S Bethell[1], Jonathan Neville[1], Mark Johnson[2, 3], Joanne Turnbull[4] and Nigel J Hall[1]

Supplementary methods

Literature search

Medline, CINAHL, Embase and the Cochrane library were searched on 6th March 2023 for Congenital duodenal obstruction OR duodenal atresia OR duodenal stenosis OR annular pancreas OR duodenal web AND transanastomotic tube OR transanastomotic tube OR nasojejunal tube OR enteral OR feeding. Duplicates were excluded. References of included studies were screened for additional studies meeting the inclusion criteria.

Search strategy

Search: **((congenital duodenal obstruction) OR (duodenal atresia) OR (duodenal stenosis) OR (annular pancreas) OR (duodenal web)) AND ((trans-anastomotic tube) OR (transanastomotic tube) OR (nasojejunal tube) OR (enteral) OR (feeding))** Sort by: **Most Recent**

((("congenital"[MeSH Subheading] OR "congenital"[All Fields] OR "congenitally"[All Fields]) AND ("duodenal obstruction"[MeSH Terms] OR ("duodenal"[All Fields] AND "obstruction"[All Fields]) OR "duodenal obstruction"[All Fields])) OR ("familial duodenal atresia"[Supplementary Concept] OR "familial duodenal atresia"[All Fields] OR "duodenal atresia"[All Fields]) OR ("familial duodenal atresia"[Supplementary Concept] OR "familial duodenal atresia"[All Fields] OR "duodenal stenosis"[All Fields]) OR ("annular pancreas"[Supplementary Concept] OR "annular pancreas"[All Fields] OR "annular pancreas"[All Fields]) OR (("duodenitis"[MeSH Terms] OR "duodenitis"[All Fields] OR "duodenum"[MeSH Terms] OR "duodenum"[All Fields] OR "duodenal"[All Fields]) AND "web"[All Fields])) AND (("trans-anastomotic"[All Fields] AND "tube"[All Fields]) OR ("transanastomotic"[All Fields] AND "tube"[All Fields]) OR ("nasojejunal"[All Fields] AND "tube"[All Fields]) OR ("enteral"[All Fields] OR "enterally"[All Fields]) OR ("feeding"[All Fields] OR "feedings"[All Fields] OR "feeds"[All Fields]))

Translations

congenital: "congenital"[Subheading] OR "congenital"[All Fields] OR "congenitally"[All Fields]

duodenal obstruction: "duodenal obstruction"[MeSH Terms] OR ("duodenal"[All Fields] AND "obstruction"[All Fields]) OR "duodenal obstruction"[All Fields]

duodenal atresia: "Familial duodenal atresia"[Supplementary Concept] OR "Familial duodenal atresia"[All Fields] OR "duodenal atresia"[All Fields]

duodenal stenosis: "Familial duodenal atresia"[Supplementary Concept] OR "Familial duodenal atresia"[All Fields] OR "duodenal stenosis"[All Fields]

annular pancreas: "Annular pancreas"[Supplementary Concept] OR "Annular pancreas"[All Fields] OR "annular pancreas"[All Fields]

duodenal: "duodenitis"[MeSH Terms] OR "duodenitis"[All Fields] OR "duodenum"[MeSH Terms] OR "duodenum"[All Fields] OR "duodenal"[All Fields]

enteral: "enteral"[All Fields] OR "enterally"[All Fields]

feeding: "feeding"[All Fields] OR "feedings"[All Fields] OR "feeds"[All Fields]

Study selection and data extraction

Studies were included if they compared use of TAT feeding in CDO versus no TAT. Only English language papers were included and conference proceedings without peer review were excluded. Abstracts were independently screened by two researchers (GB and JN) to decide whether a full text review was required. Conflicts were resolved by the senior author. Full texts were read by 2 authors (GB and JN) and included if they met the inclusion criteria.

Studies with duplicated participants and those containing no comparative outcomes were excluded from quantitative synthesis.

Data synthesis

Where studies reported only median and range, an estimation of mean and standard deviation were calculated.(1, 2) Statistical analysis and forest plot creation took place using Review Manager version 5.4 (Cochrane, Nordic Cochrane Centre, Copenhagen, Denmark). Two-sided random effects modelling was used for comparison between TAT and no TAT use and measures of effect were risk difference for categorical outcomes given that events were generally rare(3) whilst mean difference was used for continuous outcomes, both reported with 95% confidence intervals (CI). Where an event was not possible in both groups and hence there was no comparator (TAT related complication) a one-sided random effects model was used and reported as incidence with 95% CI.(4) Where meta-analysis was not possible due to zero-events a raw incidence was reported. Heterogeneity was assessed using the I^2 statistic.

Summary of findings

Grades of recommendation, assessment, development and evaluation (GRADE) guidelines were used to produce a summary table with summary data, magnitude of effect of interventions and certainty of evidence.(5)

References

1. Luo D, Wan X, Liu J, Tong T. Optimally estimating the sample mean from the sample size, median, mid-range, and/or mid-quartile range. Stat Methods Med Res. 2018;27(6):1785-805.
2. Wan X, Wang W, Liu J, Tong T. Estimating the sample mean and standard deviation from the sample size, median, range and/or interquartile range. BMC Med Res Methodol. 2014;14:135.
3. Eaton S, Hall NJ, Pierro A. Zero-total event trials and incomplete pyloromyotomy. J Pediatr Surg. 2009;44(12):2434-5; author reply 5-6.
4. Hall NJ, Jones CE, Eaton S, Stanton MP, Burge DM. Is interval appendicectomy justified after successful nonoperative treatment of an appendix mass in children? A systematic review. J Pediatr Surg. 2011;46(4):767-71.
5. Guyatt GH, Oxman AD, Schunemann HJ, Tugwell P, Knottnerus A. GRADE guidelines: a new series of articles in the Journal of Clinical Epidemiology. J Clin Epidemiol. 2011;64(4):380-2.
